# Integration of Hyperspectral Imaging and Deep Learning for Discrimination of Fumigated Lilies and Prediction of Quality Indicator Contents

**DOI:** 10.3390/foods14050825

**Published:** 2025-02-27

**Authors:** Pengfei Zhang, Youyou Wang, Binbin Yan, Xiufu Wang, Zihua Zhang, Sheng Wang, Jian Yang

**Affiliations:** 1 Dexing Research and Training Center of Chinese Medical Sciences, Dexing 334220, China; zpfei588@163.com (P.Z.);; 2 Jiangxi Province Key Laboratory of Sustainable Utilization of Traditional Chinese Medicine Resources, Institute of Traditional Chinese Medicine Health Industry, China Academy of Chinese Medical Sciences, Nanchang 330115, China; 3 State Key Laboratory for Quality Ensurance and Sustainable Use of Dao-di Herbs, National Resource Center for Chinese Materia Medica, China Academy of Chinese Medical Sciences, Beijing 100700, China; 4 Dexing Traditional Chinese Medicine Industry Development Service Center, Dexing 334220, China

**Keywords:** lily, hyperspectral imaging, nutrient content, deep learning

## Abstract

The lily, valued for its edibility and medicinal properties, is rich in essential nutrients. However, storage conditions and sulfur fumigation during processing can degrade key nutrients like polysaccharides, phenols, and sulfur dioxide. To address this, we applied a deep learning model combined with hyperspectral imaging for the rapid prediction of nutrient quality. The CLSTM (convolutional neural network–long short-term memory) model, utilizing variable combination population analysis (VCPA) for wavelength selection, effectively differentiated sulfur fumigation patterns in lilies. In terms of nutrient content prediction, the CLSTM model combined with full-wavelength data demonstrated superior performance, achieving an R^2^ value of 0.769 for polysaccharides and 0.699 for total phenols. Additionally, the CLSTM model combined with IRF-selected characteristic wavelengths exhibited remarkable performance in predicting sulfur dioxide content, with an R^2^ value of 0.755. These findings highlight the potential of hyperspectral imaging and the CLSTM model in enhancing the quality assessment and ensuring the nutritional integrity of lily products.

## 1. Introduction

The lily, the bulb derived from the perennial herb of the Liliaceae family [1], exhibits commendable culinary and medicinal attributes. Lily bulbs are rich in polysaccharides, phenols, starch, and various nutrients, imparting therapeutic functions such as cough relief, lung moisturization, and pain alleviation [2]. Beyond its medicinal qualities, the lily holds ornamental and culinary value [3]. The historical roots of lily consumption trace back to 960–900 B.C., evidenced by the Chinese tradition of consuming the bulbs of lily plants, known as ’Baihe’. The cultivation of edible lilies dates back to the Ming Dynasty, boasting a history spanning over 500 years to the present day [4]. Chemicals extracted from lily bulbs, particularly polysaccharides, demonstrate noteworthy capabilities in DPPH radical scavenging, hydroxyl radical scavenging, chelating activity, and total antioxidant capacity [5]. Polyphenolic compounds extracted from lilies present potential as an alternative adjuvant therapy for the development of functional foods aimed at inhibiting obesity and related diseases, such as metabolic syndrome and nonalcoholic fatty liver [6]. Studies underscore the promising application prospects of lilies in both the food and medicine fields.

Despite these benefits, the preservation and quality assessment of lily products remain a challenge. Lily samples are prone to enzymatic browning due to elevated water content and high activities of polyphenol oxidase (PPO) and peroxidase (POD), resulting in a significant decline in lily quality [7]. To counteract this, employing dehydration and fumigation has proven to be an effective and widely adopted pretreatment method to mitigate enzymatic browning, thereby extending the shelf life and preserving the quality of lilies. However, the detection of sulfur fumigation residues, especially in cases of excessive sulfur application, poses risks to product quality and human health. Recognizing the potential hazards to the safety and efficacy of sulfur-fumigated herbal medicines, the China Food and Drug Administration (CFDA) has enacted regulations discouraging sulfur fumigation in favor of non-sulfuration processes. Despite these regulations, some manufacturers, influenced by production conditions and economic considerations, persist in utilizing sulfur fumigation in the primary processing of lilies [8,9,10].

Given these challenges, accurately assessing the quality of lilies—particularly with respect to polysaccharide, polyphenol, starch, and sulfur dioxide content—is critical. Polysaccharides are traditionally determined through high-performance liquid chromatography (HPLC) [11], total phenols through ultra-performance liquid chromatography–quadrupole/time-of-flight mass spectrometry (UPLC-Q-TOF/MS) [12], starch content using near-infrared spectroscopy (NIR) [13], and sulfur dioxide content employing ultra-performance liquid chromatography–quadrupole/time-of-flight mass spectrometry (UHPLC-QTOF-MS/MS) [14]; these are effective but time-consuming, costly, and often destructive. This has prompted the need for more efficient, non-destructive approaches.

Hyperspectral imaging (HSI), which combines spectroscopy and imaging, has emerged as a powerful tool for food quality evaluation. HSI not only provides detailed spectral data that reflect a product’s internal physicochemical characteristics, but it also enables rapid, non-destructive analysis [15,16]. Recent studies have demonstrated the potential of HSI in detecting sulfur-fumigated products and evaluating quality indicators, such as total phenolics, polysaccharides, and starch. In the realm of food nutrients, Near-Infrared Hyperspectral Imaging (NIR-HSI) has successfully determined total phenolics in dried black goji berries (*Lycium ruthenicum* Murr.) [17], pectin polysaccharides in mulberries [18], and starch content in adulterated fresh cheese [19], yielding noteworthy research outcomes. However, there remains a limited number of studies on the discrimination of sulfur-fumigated products using hyperspectral imaging technology. Liu et al. [20] demonstrated that hyperspectral imaging technology can rapidly and non-destructively inspect Flos Lonicerae fumigated with varying sulfur concentrations. He et al. [21] proposed HSI as a promising technique for the online visualization and monitoring of SO_2_ residual content in Fritillaria thunbergii Bulbus. Additionally, Zhang et al. [22] illustrated hyperspectral imaging’s capability to distinguish between sulfur-fumigated and sun-dried traditional Chinese herbal Radix paeoniae alba. However, while HSI’s applications in other food products are well documented, there is a noticeable gap in studies focusing on the non-destructive assessment of sulfur-fumigated lilies.

To enhance the predictive capabilities of HSI, advanced modeling techniques are needed. Deep learning, a subset of machine learning, has shown great promise in processing complex datasets. Convolutional neural networks (CNNs) and long short-term memory networks (LSTMs) are particularly well suited for handling high-dimensional spectral data [23,24,25]. These models extract hierarchical features, improve prediction accuracy, and reduce human intervention in parameter selection [26,27]. For instance, Liu et al. [25] applied CNN to detect and analyze complex food matrices, Tian et al. [23] established an LSTM model to estimate wheat yield in the Guanzhong Plain, and Liu et al. achieved accurate prediction of salmon freshness under temperature fluctuations using a convolutional neural network–long short-term memory (CLSTM) model [28]. By incorporating deep learning approaches—such as convolutional LSTM (CLSTM) networks—into hyperspectral analysis, it becomes possible to achieve more reliable predictions of quality indicators while streamlining the assessment process.

Despite these advances, the integration of HSI and deep learning to predict the nutrient content of sulfur-fumigated lilies remains largely unexplored. This study seeks to address this gap by proposing a novel, rapid, and non-destructive approach that combines HSI with deep learning. Our approach aims to improve the accuracy, efficiency, and applicability of quality assessment techniques for lily products, ultimately enhancing their safety and nutritional value. More specifically, the objectives are as follows: (1) To establish a hyperspectral combined with a deep learning model for the rapid discrimination of sulfur fumigation; (2) to compare the best preprocessing and modeling methods for the dataset across several models, including SVM, CNN, LSTM, and CLSTM, in conjunction with preprocessed data and identify the most suitable models; (3) to determine the accuracy of different modeling methods based on full wavelengths and eigenbands and select the best method for eigenband selection; (4) to extract optimal wavelengths and construct a simplified model; and (5) to employ the best prediction model for the classification and prediction of the nutrient content of lilies.

## 2. Materials and Methods

### 2.1. Sample Preparation

The lily samples utilized in this study were sourced from Longshan and Shaoyang counties in Hunan Province, China, Yichang City in Hubei Province, China, Lanzhou City in Gansu Province, China, and Yuncheng City in Shanxi Province, China, with 100 samples collected from each origin, amounting to a total of 500 sample batches. For comparative analysis, the collected samples underwent a thorough process that included washing, removal of scaly leaves, and gentle scalding in boiling water. Subsequently, half of the fresh lilies were naturally sun-dried, while the other half underwent drying through sulfur fumigation. The sulfur fumigation process closely resembled the methods employed by farmers or producers [29]. During sulfur fumigation, lily samples were positioned in the upper layer of an airtight chamber, and sulfur powder was ignited on the chamber’s bottom cloth. This process released sulfur dioxide into the upper chamber, allowing it to permeate the lily samples, which were then fumigated and dried. Professor Jian Yang from the Institute of Traditional Chinese Medicine, Chinese Academy of Traditional Chinese Medicine, meticulously identified all lily samples.

### 2.2. Data Acquisition for Hyperspectral Imaging Systems

Hyperspectral images of lily samples were captured using a visible and short-wave/long-wave infrared hyperspectral imaging system (HySpex VNIR-1800/HySpex SWIR 384, Norsk Elektro Optikk, Oslo, Norway). This system comprises two lenses covering wavelengths from 350 to 1100 nm (VNIR) and 950 to 2550 nm (SWIR), accompanied by two 150 W tungsten bromide lamps (H-LAM, Norsk Elektro Optikk, Oslo, Norway) as light sources, a sample conveyor, and a computer for data collection. The two lamps were positioned at an angle of incidence of 45°. Exposure times for the two lenses, VNIR and SWIR, were set at 0.0035 s and 0.0045 s, respectively. The distance between the sample and the lenses was maintained at 32 cm, while the conveyor belt operated at a speed of 2.5 mm/s.

### 2.3. Hyperspectral Image Region Extraction and Calibration

In order to mitigate the impact of instrument and environmental factors on the sample data, wavelengths ranging from 410 to 990 nm and 990 to 2500 nm were meticulously combined manually, resulting in 396 wavelengths considered to be valid data. This combination was performed while considering noise fluctuations at the edge wavelengths. The hyperspectral images obtained were in their raw form and subsequently processed on a computer for further correction using RAD (HySpex-1600) correction software, employing black-and-white plate correction. A Spectralon^®^ white reference panel with a known reflectance of 99% was used as the white reference material to ensure accuracy. The correction equation (Equation (1)) is as follows:

(1) $$R=\frac{R_{O}-R_{b}}{R_{w}-R_{b}}$$

R: Corrected hyperspectral image.

*Ro*: Original hyperspectral image.

*Rw*: Reflectivity of white plate.

*Rb*: Reflectivity of black plate.

After image acquisition and calibration, the regions of interest were manually extracted using ENVI 5.3 software (Research Systems Inc., Boulder, CO, USA) to delineate the samples from the background through image segmentation. For this study, the entire lily sample was designated as the region of interest, and the spectral data within each region of interest was extracted, representing the average spectrum of the entire sample.

### 2.4. Analysis of Nutrient Index Composition and Sulfur Dioxide Content

#### 2.4.1. Polysaccharide Content Evaluation

The total polysaccharide content was determined using the method outlined in the Total Polysaccharide Extraction Kit (YX-W-ZDT, HEPENGBIO, Shanghai, China), which is based on established protocols for polysaccharide quantification [30]. Standards were prepared at concentrations of 1 mg/mL, 0.5 mg/mL, 0.25 mg/mL, 0.125 mg/mL, and 0.0625 mg/mL. Absorbance values (A) were measured at 490 nm, and a standard curve was constructed based on the extraction method. Following the kit’s specifications, a 0.05 g sample of lily powder was prepared, and the polysaccharide content was measured using an enzyme standardization instrument. The lily polysaccharide content was calculated according to the following formula in Equation (2):

(2) $$M_{1}=\frac{5\times Y}{W}$$

*M*_1_: total polysaccharide content (mg/g).

*Y*: sample polysaccharide concentration (mg/mL), calculated from the standard curve.

*W*: sample mass (g).

#### 2.4.2. Evaluation of Total Phenolic Content

The total phenol content was determined using the method outlined in the Total Phenol Extraction Kit (BA1506, Saint-Bio, Shanghai, China). Standards were prepared at concentrations of 0.16 mg/mL, 0.08 mg/mL, 0.04 mg/mL, 0.02 mg/mL, 0.01 mg/mL, and 0.005 mg/mL. Absorbance values (A) were measured at 760 nm, and a standard curve was constructed based on the extraction method. Following the kit’s specifications, a 0.1 g sample of lily powder was prepared, and the polyphenol content was measured using an enzyme standardization instrument. The lily polyphenol content was calculated according to the following formula in Equation (3) [31]:

(3) $$M_{2}=\frac{2.5\times X}{W}$$

*M*_2_: Total phenol content (mg/g).

*X*: sample total phenol concentration (mg/mL), calculated from the standard curve.

*W*: sample mass (g).

#### 2.4.3. Evaluation of Sulfur Dioxide Content

The residues of sulfur dioxide in lily samples were determined using the acid-base titration method outlined in the Chinese Pharmacopoeia. Initially, approximately 10 g of lily powder was precisely weighed and placed in a two-necked round-bottom flask. Subsequently, 300~400 mL of water was added, and the reflux condenser switch was activated to provide water. The upper port of the condenser was connected to a rubber air guide tube at the bottom of a 100 mL conical flask. A 50 mL 3% hydrogen peroxide solution was added to the conical flask as the absorbent solution. Prior to use, 3 drops of methyl red ethanol solution indicator (2.5 mg/mL) were added to the absorbent solution, and titration with 0.01 mol/L sodium hydroxide titrant was conducted until a yellow color was achieved. Following this, nitrogen gas was introduced, and the gas flow rate was adjusted to approximately 0.2 L/min using a flow meter. The piston of separatory funnel C was opened, allowing 10 mL of 6 mol/L hydrochloric acid solution to flow into the distillation flask. The solution in the two-necked flask was immediately heated to boiling and maintained at a slight boil. After boiling for 1.5 h, the heating was ceased. Subsequently, the absorbent solution was cooled, placed on a magnetic stirrer with constant stirring, and titrated with 0.01 mol/L sodium hydroxide titrant until the yellow color persisted for 20 s without fading. The titration was corrected using a blank experiment. The sulfur dioxide residue in lilies was calculated according to the following formula in Equation (4):

(4) $$M_{3}=\frac{\left(A-B\right)\times c\times 0.032\times 10^{6}}{W}$$

*M*_3_: Sulfur dioxide residues (μg/g).

*A*: Volume of sodium hydroxide titrant consumed by the test solution (mL).

*B*: Volume of blank consumption of sodium hydroxide titrant (mL).

*c*: Molar concentration of sodium hydroxide titration solution (mol/L).

The value of 0.032 is the equivalent of 1 mL of sodium hydroxide titrant (1 mol/L) of SO mass of sulfur (g).

*W*: Weight of the test sample (g).

### 2.5. Chemometrics Analysis

#### 2.5.1. Support Vector Machine (SVM)

Support Vector Machine (SVM), a supervised learning algorithm, has emerged as a robust tool for addressing both classification and regression problems. SVM demonstrates its utility in the regression prediction of chemical content, showcasing a distinctive capability to handle linear and nonlinear spectral data in pattern recognition. Renowned for its excellent generalization performance and accurate prediction ability, SVM finds wide applications [32]. Presently, SVMs are adept at regression tasks through the incorporation of ε-insensitive loss functions, a form of utilization known as support vector regression.

#### 2.5.2. Deep Learning Model of Convolutional Neural Networks (CNNs)

**Figure 1:**
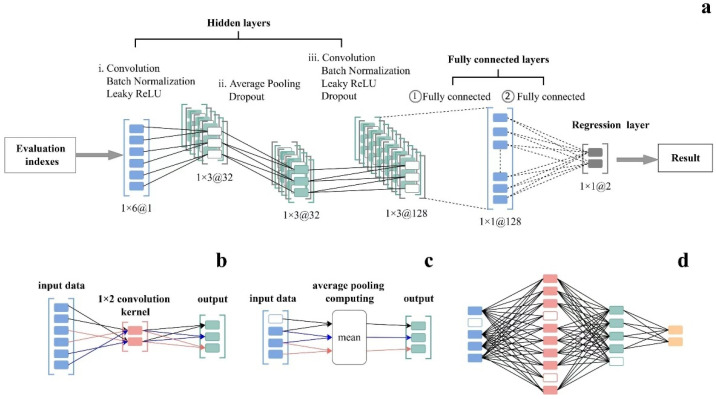
The CNN deep learning model. (**a**) Convolutional Neural Network (CNN) Architecture for Regression, (**b**) Convolution Operation with 1 × 2 Kernel, (**c**) Average Pooling Computation, (**d**) Fully Connected Neural Network Structure.

The convolutional neural network (CNN) is a well-established deep learning architecture inspired by the natural visual perception mechanisms found in living organisms [33]. The CNN deep learning model, as illustrated in Figure 1, comprises 3 hidden layers (Figure 1a), 2 fully connected layers, and 1 regression layer. In detail, the first hidden layer includes a batch normalization layer, 32 convolutional kernels, 2 convolutional layers with sliding steps, and an activation layer (Figure 1b). The second hidden layer incorporates an average pooling layer and a dropout layer to enhance the generalization of the CNN model (Figure 1c). The third hidden layer encompasses 128 convolutional kernels, a convolutional layer with sliding steps, a batch return layer, an activation layer, and an exit layer (Figure 1d). The hyperbolic tangent function is employed to activate the fully connected layer. Furthermore, the outputs of the fully connected layer and the fully connected layer are vector levels of lengths 128 and 2, respectively, with the length equal to the number of output variables.

#### 2.5.3. Long Short-Term Memory (LSTM)

**Figure 2:**
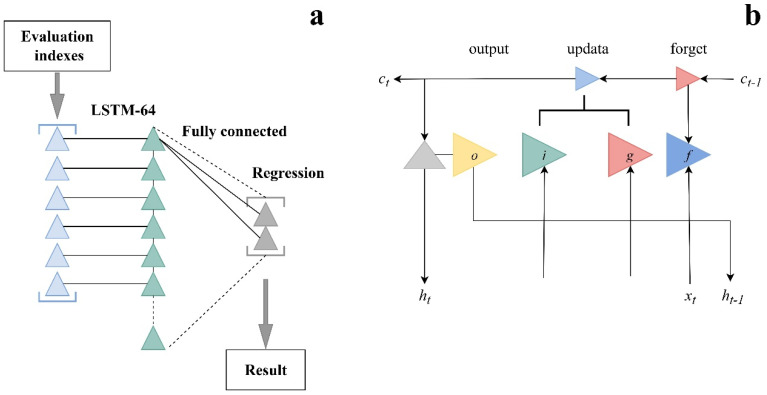
The structure of the LSTM model. (**a**) Long Short-Term Memory (LSTM) Network Architecture for Regression, (**b**) LSTM Cell Structure and Computation Flow.

LSTM, a standard type of Recurrent Neural Network (RNN), is widely utilized for learning and processing long-term information, time series data, feature extraction, and pattern recognition [34]. In this paper, building upon the fully connected layer CNN model, we propose an LSTM model with 64 hidden cells, and its detailed structure is depicted in Figure 2a. The LSTM layer involves two states: the output state (h_t_ at time step t) and the cell state (*c_t_* at time step t). Both states are controlled by the input gate (i), the forgetting gate (f), the cell candidate gate (g), and the output gate (o) (Figure 2b). The primary computation formula of the LSTM is articulated as follows in Equation (5) [35]:

(5) $$f_{t}=\sigma \left(W_{f}\left(h_{t-1},x_{t}\right)+b_{f}\right)\;i_{t}=\sigma \left(W_{i}\left(h_{t-1},x_{t}\right)+b_{i}\right)\;s_{t}=tanh\left(W_{g}\left(h_{t-1},x_{t}\right)+b_{g}\right)\;C_{t}=f_{t}c_{t-1}+i_{t}8t\;o_{t}=\sigma \left(W_{0}\left(h_{t-1},x_{t}\right)+b_{o}\right)\;h_{t}=o_{t}\tan h\left(c_{t}\right)$$

The output values of the forget gate (*f_t_*), input gate (*i_t_*), update gate (g_t_), and output gate (*o_t_*) are determined by the corresponding formulas. Here, W_f_, W_i_, W_g_, and *W*_o_ represent the weight matrices, while b_f_, b_i_, b_g_, and *b*_o_ illustrate the bias vectors. The memory cell (*c_t_*) and the sigmoid activation function (σ) are denoted by ct and σ, respectively. Additionally, the inputs of the four gates include the LSTM target value h_t−1_ at a past time step t − 1.

#### 2.5.4. Module Combination of CNN-LSTM (CLSTM)

**Figure 3:**
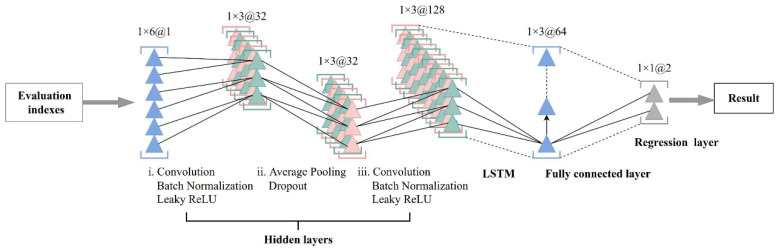
The structure of the CLSTM model.

In the CLSTM model, the configuration of the convolutional layer mirrors that of the CNN model, with the addition of an LSTM layer after the convolutional layer, thereby replacing the first fully connected layer. The convolutional layer serves as a feature extractor for the original data, and the output is then fed into the LSTM to generate predictions. This methodology is commonly referred to as CLSTM in the literature. Figure 3 illustrates the detailed structure of the proposed CLSTM model, incorporating spatio-temporal fusion information. Both the CNN and CLSTM models share identical configurations for the convolutional layer, and the LSTM layer configuration remains consistent between the LSTM and CLSTM models to ensure a fair performance evaluation (Table S1). Moreover, for the training set, all three deep learning models comprise the same modules and possess identical settings to facilitate a fair comparison (Table S2).

In the recognition of sulfur fumigation patterns, the four aforementioned models, combined with the selection of hyperspectral full wavelength and feature wavelength, were employed for dichotomous recognition. For nutrient content prediction, widely used metrics such as mean absolute error (MAE), Pearson correlation coefficient (*R*), and root mean square error (RMSE) were utilized to evaluate the performance of the models, as defined below (Equation (6)):

(6) $$RMSE={\left[\frac{\sum_{k}^{N}=1{\left(S_{k}^{\prime }-S_{k}\right)}^{2}}{N}\right]}^{1/2}\;MAE=\frac{\left(\sum_{k}^{N}=1\left|S_{k}^{\prime }-S_{k}\right|\right)}{N}\;R=\frac{\left[\sum_{k}^{N}=1\left(S_{k}^{\prime }-b\right)\times \left(S_{k}-a\right)\right]}{{\left[\sum_{k}^{N}=1{\left(s^{\prime }-b\right)}^{2\times \sum_{k=1}^{N}{\left(S_{k}-a\right)}^{2}}\right]}^{1/2}}$$

where *N* represents the number of samples, *s_k_* represents the actual value, *s’_k_* represents the predicted value, *a* represents the mean of the actual values, and *b* represents the predicted values.

### 2.6. Optimal Wavelength Selection Methods

Variable (wavelength or feature) selection techniques have become a crucial step in analyzing datasets with a large number of variables and relatively small samples. Variable selection plays a pivotal role in multivariate analysis by eliminating irrelevant information and extracting important details related to sample attributes. In this paper, two algorithms, namely interval random frog (iRF) and variable combination population analysis (VCPA), are employed to extract the optimal wavelengths.

The iRF (interval random frog) algorithm utilizes the random frog algorithm, which is a reversible jump Markov chain Monte Carlo algorithm initially designed for gene selection [36]. This algorithm explores the model space by transitioning between various models within and across dimensions. It then generates a pseudo-MCMC chain, which is employed to calculate the selection probability for each variable. Subsequently, variables are selected based on the ranking of all variables [37].

Variable combination population analysis (VCPA) [38] is an innovative variable selection method that takes into account potential interactions between variables through random combinations. VCPA employs an exponentially decreasing function (EDF) based on the simple and effective “survival of the fittest” principle of Darwinian evolution to determine the number of variables to be retained.

## 3. Results

### 3.1. Identification of Sulfur-Fumigated Lilies by HSI Wavelength-Based Chemome-Tric Model

**Figure 4:**
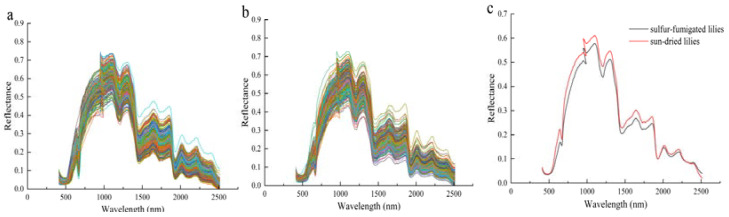
(**a**) Hyperspectral image of dried lily, (**b**) hyperspectral image of sulfur-fumigated lily, and (**c**) average spectra of dried and sulfur-fumigated lilies.

Due to noise caused by the detector, significant spectral noise is observed at both the head and tail of the spectrum. Therefore, only spectra in the range of 400–2500 nm were used for analysis. Figure 4a shows the spectra of dried lily extracts, while Figure 4b displays the spectra of sulfur-fumigated lily extracts. Figure 4c presents the average spectra of both sulfur-fumigated and sun-dried samples. Minor differences were observed in the average spectra of samples under different treatments, indicating the impact of sulfur fumigation on the samples. The identification of fumigated lilies using the chemometric model is presented in Table 1. In the results of full-wavelength detection, the support vector machine (SVM) model exhibits a training set accuracy of 79.4% and a test set accuracy of 74.6%. Among the deep learning models, the convolutional neural network (CNN) model achieves a training set accuracy of 87.4% and a test set accuracy of 79.3%. The long short-term memory (LSTM) model attains a training set accuracy of 89.4% and a test set accuracy of 84.0%. When the CNN model is combined with the LSTM model, the training set accuracy increases to 92.9%, and the test set accuracy reaches 90.7% (Table 1). The results indicate that the combination of CNN and LSTM enhances both the training and test set accuracy by approximately 10%, suggesting improved model accuracy and discrimination capability between sulfur-smoked and non-sulfur-smoked lilies. The integration of CNN and LSTM, covering spatial and temporal data information, ensures adaptability for complex nonlinear systems, providing robust reasoning and expression of the topological relationships between variables. In conclusion, the hyperspectral imaging (HSI) technique combined with the CLSTM deep learning model demonstrates significant potential for application in sulfur fumigation recognition.

### 3.2. Results from Effective Wavelengths Group

Through feature band selection using interval random frog (iRF) and variable combination population analysis (VCPA), the training and test sets of the SVM, CNN, LSTM, and CLSTM models exhibit improvements compared to the full band. In the SVM model, both the training set and test set show an improvement of about 12%. In the deep learning models CNN and LSTM, the training set exceeds 93%, and the test set is above 82%. When the CNN and LSTM combine in the CLSTM model, both the training set and test set exceed 92% in the iRF feature band, reaching 97% in the VCPA feature band (Table 1). The feature wavelengths identified by VCPA are between 1200 nm and 1400 nm, possibly indicating a reduction in chemical components (polysaccharides, starch, etc.) due to sulfur dioxide residue from sulfur fumigation. These results highlight that feature band selection can enhance the model's accuracy. Specifically, the combination of the VCPA feature band with deep learning CLSTM discrimination accurately identifies sulfur-fumigated lily samples.

### 3.3. Prediction of the Content of Three Chemical Components

#### 3.3.1. Results from Full-Wavelengths Group

**Figure 5:**
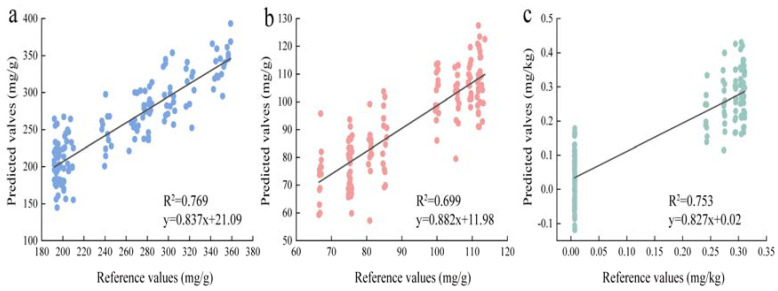
Reference versus predicted values for three nutrient contents from the best−performing model groups. (**a**–**c**): The ALSTM model based on full wavelengths exhibited the best performance in predicting polysaccharide, total phenol, and SO_2_ content. R^2^: the square of the curve correlation coefficient.

The results of nutrient content prediction using HSI full wavelengths are presented in Table 2. The models’ correlation coefficient (R), mean absolute error (MAE), and root mean square error (RMSE) were employed as indicators to determine the optimal pretreatment method. Higher R-values and lower MAE and RMSE values suggest more accurate prediction results. The comprehensive analysis of the three chemical substances using full-wavelength detection revealed that, in the prediction of polysaccharide content, the CLSTM group outperformed the SVM, CNN, and LSTM groups, exhibiting higher R-values and lower MAE and RMSE values. The CLSTM model demonstrated the best prediction results, with approximately a 20% decrease in MAE and RMSE values and a 5% improvement in R-values compared to the other models. Also, the regression results based on the reference and predicted polysaccharide content values (Figure 5a) show both the slope and R^2^ values are above 0.76. Similarly, in the prediction of total phenol and sulfur dioxide content, the CLSTM group exhibited higher R-values and lower MAE and RMSE values compared to the SVM, CNN, and LSTM groups. Specifically, for total phenol, the CLSTM model achieved a 7.4% improvement in R-value, along with a 25–37% decrease in MAE and RMSE values. In addition, the regression results based on the reference and predicted total phenol content values (Figure 5b) show both the slope and R^2^ values are above 0.69. For sulfur dioxide, the R-value improved by approximately 7%, and MAE and RMSE decreased by around 25–32%. Also, the regression results based on the reference and predicted SO_2_ content values (Figure 5c) show both the slope and R^2^ values are above 0.75. Overall, the CLSTM model demonstrated superior performance in predicting the content of all three compounds compared to the other three models.

#### 3.3.2. Results from Effective Wavelengths Group

Wavelength selection is a critical step in enhancing the prediction performance of spectral data. The interval random frog (iRF) algorithm, coupled with variable combination population analysis (VCPA) feature wavelength selection, was employed to identify informative feature wavelengths for the three compounds in lilies. This aimed to reduce non-informative spectral data and enhance model performance. The results of nutrient content prediction using HSI characteristic wavelengths are presented in Table 2. In the iRF analysis, the CLSTM group exhibited higher R-values and lower mean absolute error (MAE) and root mean square error (RMSE) values in polysaccharide prediction compared to the SVM, CNN, and LSTM groups. When compared to the full-wavelength group, the CLSTM group showed higher R-values than the SVM and CNN groups, with lower MAE and RMSE values. The R-values, MAE, and RMSE values were similar to those of the LSTM full-wavelength group, indicating that the combination of the CLSTM group with iRF feature band selection achieves comparable results to the combination of the LSTM group with the full-wavelength band. Similarly, for both total phenol and sulfur dioxide predictions, the CLSTM model demonstrated higher R-values and lower MAE and RMSE values than the SVM, CNN, and LSTM groups. These results suggest that the combination of CLSTM and iRF provides superior performance compared to other models for predicting total phenol and sulfur dioxide content.

The results obtained from the variable combination population analysis (VCPA) calculations demonstrated that the combination of CLSTM and VCPA exhibited higher R-values compared to the SVM, CNN, and LSTM groups for the prediction of total phenol and SO_2_. Additionally, it showed lower mean absolute error (MAE) and root mean square error (RMSE) values than the SVM, CNN, and LSTM groups. Moreover, the combination of CLSTM and VCPA showcased R-values that were approximately equal and lower MAE and RMSE values compared to the combination of CLSTM and iRF. These findings indicate that the combination of CLSTM and VCPA offers enhanced accuracy in predicting total phenols and sulfur dioxide content.

## 4. Discussion

### 4.1. Comparison of Discriminatory Results Between Fumigated and Non-Fumigated Lilies

In this study, the full-wavelength group’s test results revealed that the accuracy of the SVM, CNN, and LSTM model group on the test set ranged from 75% to 84%. Through model combination (CLSTM group), the model accuracy of the test set reached 90%, showcasing an improvement in model discrimination accuracy by about 6% compared to CNN or LSTM. This indicates a significant enhancement in prediction accuracy through model combination. In the feature wavelength test results, the accuracy of both iRF and VCPA feature wavelengths improved relative to the full wavelength, and the selection of both feature wavelengths and combined models (CLSTM group) improved compared to the SVM, CNN, and LSTM groups. The iRF feature wavelengths combined with CLSTM models improved accuracy by about 2%, and the accuracy of VCPA feature wavelengths combined with the CLSTM model improved by about 7%, reaching 97.3%. These results demonstrate that the VCPA feature band combined with the CLSTM model effectively distinguishes fumigated lilies from non-fumigated lilies.

### 4.2. Comparison of Chemical Index Prediction Based on Full Wavelength and Characteristic Wavelength

In this study, the examination of three indicators—polysaccharide, total phenol, and sulfur content of lily—employed four models, namely SVM, CNN, LSTM, and CLSTM. The CLSTM combined model yielded predicted R^2^-values, MAE values, and RMSE values for polysaccharide content as 0.769, 19.63, and 25.23, respectively; for total phenol content, the values were 0.699, 6.30, and 9.34; and for sulfur dioxide content, the values were 0.753, 0.057, and 0.072. The R^2^ values for the three detection metrics surpassed those of the SVM, CNN, and LSTM models, and the MAE and RMSE values were lower than those of the SVM, CNN, and LSTM models. These results indicate that the combined CLSTM model is more accurate in detecting the three chemical content indicators in full-wavelength detection. In the detection of chemical indicators in the characteristic wavelength group, the R-values of the SVM model combined with the VCPA characteristic wavelength for the prediction of polysaccharide content were 0.702 and 0.691 for the prediction of total phenol content, which were higher than those of the CNN, LSTM, and CLSTM combined model groups combined with the iRF or VCPA characteristic wavelength. However, in the prediction of sulfur dioxide content, the R-values of the CLSTM model combined with iRF for sulfur dioxide detection were 0.755, which was higher than the combination of other models with feature wavelengths. These results indicate that the SVM model combined with the iRF feature wavelength was more accurate in predicting polysaccharides and total phenols, and the CLSTM model combined with the iRF feature wavelength was more accurate in predicting sulfur dioxide.

However, in the prediction of polysaccharide content, the CLSTM model combined with the iRF characteristic wavelength had an R^2^ value of 0.659. For the prediction of total phenol content, the CLSTM model combined with the VCPA characteristic wavelength resulted in an R^2^ value of 0.677. Similarly, in the prediction of sulfur dioxide content, the CLSTM model combined with the VCPA characteristic wavelength yielded an R^2^ value of 0.717. These values show significant differences compared to the predictive performance obtained using the optimal model with either full wavelengths or characteristic wavelengths.

## 5. Conclusions

In conclusion, this study demonstrates the effectiveness of combining hyperspectral imaging with deep learning to distinguish between fumigated and non-fumigated lilies. The CLSTM model, enhanced by VCPA wavelength selection, delivered robust predictions for polysaccharides, total phenols, and sulfur dioxide, achieving higher accuracy than full-wavelength approaches. This confirms the utility of the method in accurately assessing key nutrient indicators in fumigated samples. Key takeaways include the model’s superior predictive performance, which highlights its potential as a rapid, nondestructive, and reliable tool for quality evaluation. The ability to identify sulfur fumigation patterns and nutrient levels provides a solid foundation for improving lily product safety and nutritional value. Future research could focus on adapting this approach to a real-time monitoring system. This would involve developing portable hyperspectral devices integrated with deep learning algorithms capable of in situ analysis. Additionally, exploring the generalizability of the method to other agricultural and medicinal products could further validate its broader applicability.

## Figures and Tables

**Table 1:** Chemometric modeling for discrimination of sulfur-fumigated lilies.

Groups	SVM		CNN		LSTM		CLSTM	
	Train (%)	Test (%)	Train (%)	Test (%)	Train (%)	Test (%)	Train (%)	Test (%)
Full	79.4	74.6	87.4	79.3	89.4	84.0	92.9	90.7
iRF	82.9	84.7	92.9	88.7	92.6	86.7	94.0	92.7
VCPA	87.4	86.7	94.0	88.0	93.4	82.7	97.1	97.3

**Table 2:** Predictive modeling of lily nutrient content using chemometric approaches.

Modules	Groups	Total Polysaccharide			Total Phenol			SO_2_		
		R^2^	MAE	RMSE	R^2^	MAE	RMSE	R^2^	MAE	RMSE
SVM	Full	0.477	35.38	44.90	0.531	9.33	12.52	0.643	0.084	0.095
	iRF	0.591	28.44	35.41	0.640	8.27	10.55	0.440	0.0886	0.107
	VCPA	0.702	24.78	30.30	0.691	7.85	9.01	0.533	0.075	0.097
CNN	Full	0.581	27.39	33.67	0.599	10.14	12.23	0.643	0.079	0.096
	iRF	0.624	27.18	34.64	0.623	9.84	12.34	0.682	0.043	0.082
	VCPA	0.524	31.11	39.00	0.561	10.26	12.67	0.712	0.068	0.089
LSTM	Full	0.692	24.33	29.03	0.591	8.53	11.19	0.694	0.063	0.080
	iRF	0.616	25.32	34.1	0.618	7.74	10.38	0.709	0.054	0.077
	VCPA	0.465	32.92	41.21	0.640	8.12	11.18	0.582	0.067	0.093
CLSTM	Full	0.769	19.63	25.23	0.699	6.30	9.34	0.753	0.057	0.072
	iRF	0.659	25.36	32.61	0.671	8.97	10.74	0.755	0.052	0.072
	VCPA	0.558	32.44	39.37	0.677	6.78	9.72	0.717	0.040	0.076

## Data Availability

The original contributions presented in this study are included in the article/Supplementary Materials. Further inquiries can be directed to the corresponding authors.

## Supplementary Materials

The following supporting information can be downloaded at: https://www.mdpi.com/article/10.3390/foods14050825/s1, Table S1: The same parameters of deep learning models for fair comparison; Table S2: The same setting in training set for fair comparison between different deep learning models.
