# Supplementary material for: Integration of Hyperspectral Imaging and Deep Learning for Discrimination of Fumigated Lilies and Prediction of Quality Indicator Contents
Source: Foods. 2025 Feb 27;14(5):825. doi: 10.3390/foods14050825 (PMC11898805; doi:10.3390/foods14050825)
Supplement: Supplementary file 1 [file foods-14-00825-s001.zip › foods-3440529-supplementary.pdf]

Supplementary Materials

Table S1. The same parameters of deep learning models for fair comparison.

Layers	Parameters		
	CNN	LSTM	CLSTM
Attention	-	-	-
Convolution	128	-	128
Batch normalization	792	-	792
Leaky ReLU	0	-	0
Average pooling	0	-	0
Dropout	0	-	0
Convolution	3104	-	3104
Batch normalization	200	-	200
Leaky ReLU	0	-	0
Dropout	0	-	0
Fully connected	102464	-	-
LSTM	-	4352	8320
Fully connected	65	12673	1601
Total	106753	17025	15145

Table. S2. The same setting in training set for fair comparison between different deep learning models

Parameters	Set	Parameters	Set
CNN layer initializer	Kaiming	Epochs	200
LSTM layer initializer	Orthogonal	Dropout rate	0.2
Fully connected initializer	Kaiming	Bach size	40
Optimizer	Adam	Loss (Content prediction)	MSE
Learning Rate	1e-3	Loss (Origin prediction)	Cross Entropy
